# Effect of different surface treatments on surface roughness, phase transformation, and biaxial flexural strength of dental zirconia

**DOI:** 10.34172/joddd.2021.035

**Published:** 2021-08-25

**Authors:** Niknaz Yahyazadehfar, Maryam Azimi Zavaree, Sayed Shojaedin Shayegh, Mobin Yahyazadehfar, Tabassom Hooshmand, Seyed Mohammad Reza Hakimaneh

**Affiliations:** ^1^Department of Prosthodontics, Faculty of Dentistry, Shahed University, Tehran, Iran; ^2^Independent Investigator, Delaware, USA; ^3^Department of Dental Biomaterials, School of Dentistry/Research Center for Science and Technology in Medicine, Tehran University of Medical Sciences, Tehran, Iran

**Keywords:** Flexural strength, Surface properties, Y-TZP ceramic, YSGG laser

## Abstract

**Background.** Interfacial failures at the cement‒restoration interface highlights the importance of effective surface treatment with no adverse effect on the zirconia’s mechanical properties. This study aimed to determine the effect of different surface treatments on dental graded zirconia’s surface roughness and certain mechanical properties.

**Methods.** Forty sintered zirconia specimens were randomly divided into four groups (n=10): control (no surface treatment), sandblasting (SA), grinding with diamond bur (GB), and Er,Cr:YSGG laser (LS). Following surface treatment, the surface roughness and surface topography of the specimens were examined. X-ray diffraction (XRD) was conducted. In addition, the biaxial flexural strengths of specimens were evaluated. The data were analyzed using one-way analysis of variance (ANOVA) and post hoc Tukey tests; the Pearson correlation coefficient was calculated between either volumetric percentage of monoclinic phase or roughness and flexural strength of specimens (α=0.05).

**Results.** The GB group exhibited significantly greater surface roughness compared to the other groups (*P* < 0.005). The LS and control groups exhibited a significantly lower volumetric percentage of the monoclinic phase (*P* < 0.001) than the GB and SA treatments. The SA group exhibited significantly higher flexural strength than the control (*P* = 0.02) and GB groups (*P* < 0.01). Furthermore, the Weibull analysis for the LS showed higher reliability for the flexural strength than other treatments.

**Conclusion.** Er,Cr:YSGG laser treatment, with the lowest extent of phase transformation and reliable flexural strength, can be a promising choice for surface treatment of zirconia.

## Introduction

The increasing esthetic needs in dentistry have led to a focus on natural-appearing ceramic restorations. In this regard, the stabilized tetragonal zirconia (Y-TZP) exhibits some superiority over other ceramic types with improved mechanical properties, biocompatibility, and esthetic characteristics.^1-5^ This introduction explains numerous benefits, including superior mechanical properties, compared to the monolithic lithium disilicate ceramics and manufacturing thinner restorations for more conservative dental preparations.^6^ This would also lead to the possibility of new standardization and cost reduction due to the CAD-CAM processing technique.^6^ Furthermore, Y-TZP zirconia has a unique characteristic that can transform from tetragonal to monolithic phase under mechanical stresses, resulting in extra toughness and strength and hindering crack propagation by 3%‒5% volume expansion during phase transformation.^2^

Despite its robust mechanical properties, zirconia crowns clinically suffer from interfacial failures.^7^ They are commonly caused by the weak interfacial strength between the cement and ceramic crowns that cannot tolerate the mechanical stresses in the oral environment.^8^

There are different methods to improve the interfacial adhesion, and the purpose is to increase the surface area and decrease the stress levels at the interfaces.^9^ In restorative dentistry, this can be achieved by combining multiple methods, including some well-established treatments, such as grinding/abrasion with a diamond bur, sandblasting, acid etching, and silanization.^9-12^ More recently, laser irradiation, including Er,Cr:YSGG laser, has been proposed in this regard.^13,14^ Lasers can gather and concentrate high magnitudes of energy on target areas. Lasers, in some cases, induce chemical reactions that alter the shape and, in other situations, only cause physical changes. Er,Cr:YSGG laser has the potential to remove particles through a mechanism called ablation, including micro-explosions and vaporization.^15^ During vaporization, the internal pressure builds up within the tissue until the inorganic material is explosively destroyed before the melting point is reached.^13^ However, laser treatment needs special care for any use in dental crown surface treatment.

Martins et al,^16^ Kurtulmus-Yilmaz et al,^17^ Liu et al,^18^ and Kosmac et al^19^ have determined the effects of different surface treatments on the zirconia and have reported contradictory results.

. This study aimed to evaluate the effect of different surface treatments, including grinding by a bur, sandblasting, and laser irradiation, on the properties of dental graded zirconia. The null hypothesis was that these surface treatments do not affect the surface roughness, surface topography, and flexural strength of zirconia specimens.

## Methods

A pre-sintered 98-mm zirconia block (Shenzhen Upcera Dental Co., Ltd., China) was milled using a computer-aided design/manufacturing system to produce forty disk-shaped specimens with a diameter of 12 mm and a thickness of 1.2 (±0.2) mm. The disks were sintered at 1450ºC according to the manufacturer’s instructions in the furnace (Ceramill Therm; Amman Girrbach, Austria) and then polished with 600-, 800-, and 1200-grit silicon carbide papers (Struers A/S) for a minimum of 30 minutes under a 10-N load using a grinding/polishing machine (Phoenix; Beta Grinder/Polisher, Buehler, USA) at 300 rpm.^20^

The finished specimens were randomly divided into a nominally flaw-free control group evaluated directly (n = 10) and three other groups with different surface treatments as follows:

*Grinding with bur (GB):* The specimens in this group were processed using a high-speed hand tool with a diamond bur (Drendel + Zweiling Diamant GmbH Inc., Germany) (Table 1) at 200-kPa pressure for 20 seconds with back-and-forth motions under water coolant and regarded as the grinding group.

**Table 1:** Description of the materials used for the surface treatment

**Material**	**Main composition**	**Manufacturer**
Pre-sintered zirconia blanks (Yttrium partially stabilized zirconia)	Nanometer zirconia powder > 98% Fe_2_O_3_: < 0.3% Pr_2_O_3_: < 0.2% Er_2_O_3_: < 0.2% Other oxides: ≤0.5%	Shenzhen Upcera Dental Co. Ltd, China
Silicon carbide grinding paper	Silicon carbide Grit size: 600, 800, 1200	Struers A/S Inc.
Cylindrical blue-yellow band diamond rotary instrument	Diamond particles (108-120 m) and binder	Drendel + Zweiling Diamant GmbH, Germany

*Sandblasting with alumina (SA):* The specimens were subjected to sandblasting by 110-µm aluminum oxide particles with 2.5-bar pressure at a 30-mm distance for 30 seconds.

**Figure 1:**
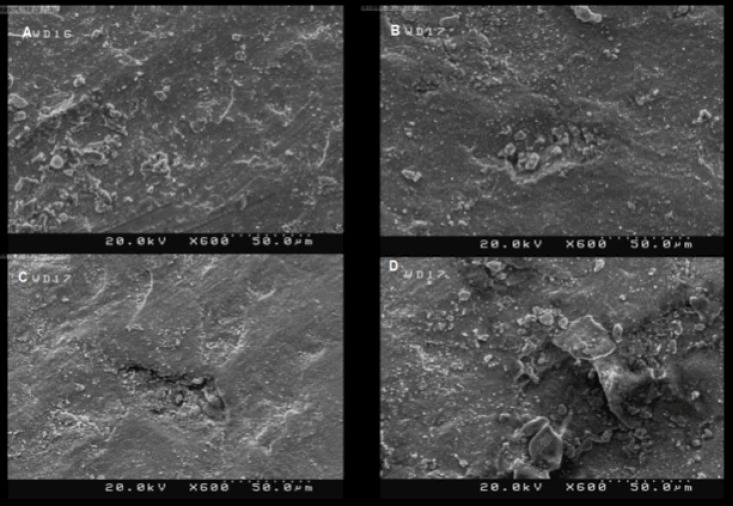
Scanning electron micrographs of Zirconia surface treated by laser at different power. (A) 1.5 (B) 2.25 W (C) 3.00 W (D) 3.75 W.

*Laser treatment (LS):* The specimens were subjected to Er,Cr:YSGG laser irradiation (iPlus, Biolase, Inc. San Clemente, California, USA) with 2940-nm wavelength fiber-optic system (1 mm in diameter) and 400-µm diameter head handpiece for 10 seconds under 80% water (32 mL/min) and 80% air settings. The laser was irradiated at a 1-mm distance from the surface in non-contact mode. The laser pulse in this study was 74 µs at 1.5-W power output and the frequency of 15 Hz. The proper power output for the laser was selected through a pilot study on four extra disc-shaped samples irradiated at 1.5, 2.25, 3, and 3.75 W (equivalent to 100, 150, 200, and 250 mJ/pulse, respectively). Then, the surface topography of the specimens was examined under a scanning electron microscope (SEM) (ZEISS DSM-960A, Germany) at ×600 magnification. There was less surface damage in the specimens treated with 1.5-W power intensity (Figure 1). The specimens were then cleaned in distilled water in an ultrasonic bath and then air-dried.

**Figure 2:**
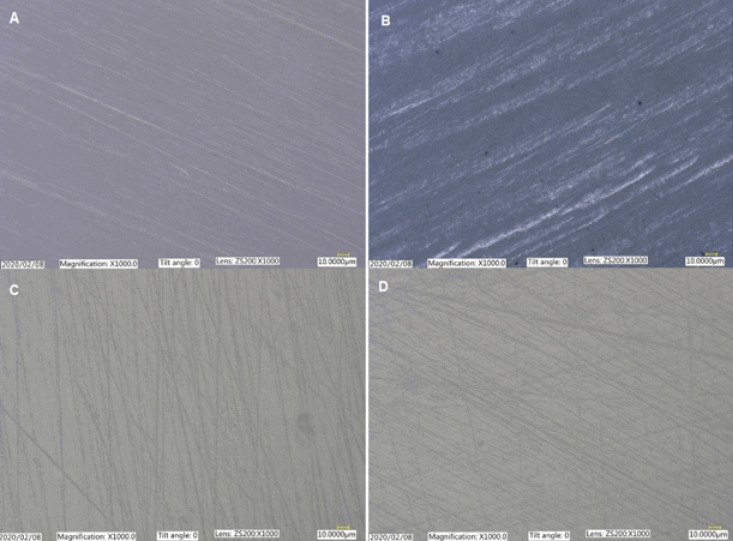
Optical micrographs of surface treated Zirconia (A) Control (B) Grinding (C) Laser (D) Sandblasting.

The average surface roughness (Ra) and peak-to-valley height (Rz) of the specimens were assessed under an optical microscope (Keyence VHX2000, USA) equipped with a ×1000 objective lens, following the ISO 25178 [ISO25178-2,2012].^21^ The images were captured at 1600×1200 pixels, which was equivalent to 400.0-300.0 μm field of view. Five measurements were performed for each specimen over a 240-μm length at a magnification of ×1000, and the means of measurements were reported as the roughness values for each specimen (Figure 2).

X-ray diffraction (XRD; Ultimate IV X-ray Diffractometer, Rigaku, Japan) was conducted to evaluate the relative percentage of the monoclinic phase on the treated specimens operated at 150 mA and 50 kV at 2Ø range, 5‒80 degrees, 0.02º step size and 50-second stop at each step. Three specimens were randomly selected from each group for this measurement. The relative percentage of monoclinic phase and phase transformation were determined from the integral intensities of the monoclinic M (-111) and M (111), and the tetragonal T (111) peaks according to the equation below^22,23^:

(1) $$X_{m}=\left[I_{m}\left(-111\right)+I_{m}\left(111\right)\right]/[\left[I_{m}\left(-111\right)+I_{m}\left(111\right)+I_{t}\left(101\right)\right]$$

(2) $$V_{m}=1.311X_{m}/\left(1+0.311X_{m}\right)$$

where X_m_ is the ratio of monoclinic peak intensity, V_m_ is the volumetric percentage of the monoclinic phase content, I_m_ (-111) and I_m_ (111) are the severity of monoclinic peak at 2Ø = 28.2°, 2Ø = 31.4°, and 1t (111) is the intensity of tetragonal phase at 2Ø = 31.1°.

The biaxial flexural strengths were evaluated using a universal testing frame (Zwick Roell, Ulm, Germany) with a 1000-N load cell. The specimens were placed on a fixture with three equidistance stainless steel spherical balls, distributed on the periphery of a 10-mm diameter circle. The load was applied at a rate of 1 mm/min to the opposite side of the treated surface by a cylindrical head piston (1.4 mm in diameter) so that the treated surface was subjected to the flexural tension (ISO6872:2008).^24^

The biaxial flexural strength was calculated using the formula below:

(3) $$S=-0.2387\frac{P\left(X-Y\right)}{d^{2}}$$

where S is the flexural strength, P is the load required for fracture, and *d* is the thickness of the specimen. *X* and *Y* were also calculated as follows:

(4) $$X=\left(1+v\right)\ln{\left(\frac{r_{2}}{r_{3}}\right)}^{2}+\left(\frac{1-v}{2}\right)\;{\left(\frac{r_{2}}{r_{3}}\right)}^{2}\;$$

(5) $$Y=\left(1+v\right)\left(1+\ln{\left(\frac{r_{1}}{r_{3}}\right)}^{2}\right)+\left(1-v\right)\;{\left(\frac{r_{1}}{r_{3}}\right)}^{2}$$

where r_1_ and r_2_ are the radii of supported and loading balls, respectively, and r_3_ is the radius of the zirconia disk. The value of 0.25 was used for the Poisson’s ratio-ѵ of dental zirconia in the equation above. In addition, the two-parameter Weibull analysis was applied to characterize the flaw size distribution of different groups in this study following the method introduced by Quinn and Quinn.^25,26^ Based on the description of the Weibull distribution, the probability of failure can be defined as:

(6) $$P_{f}=1-\text{exp}\left[-{\left(\frac{\sigma }{\sigma_{0}}\right)}^{m}\right]$$

where σ is the failure strength for each test, σ_0_ is the characteristic strength, m is the Weibull modulus, and P_f_ denotes the failure probability. Taking double algorithm of eq. 6 yields:

(7) $$lnln\left(\frac{1}{1-P_{f}}\right)=mln\sigma -ln\sigma_{0}$$

That would allow simple linear regression to calculate characteristic strength, σ_0,_ and Weibull modulus, m.

### Statistical analysis

One-way ANOVA and post hoc Tukey tests were conducted using SPSS 22 (SPSS Inc.) to compare different groups in this study regarding roughness, XRD, and flexural strength. The level of significance was set at *P* < 0.05. Moreover, Pearson’s correlation coefficient test was carried out to find potential correlations between the variables.

## Results

Figure 2 presents the optical microscope images of the surface of control and treated specimens. All the samples exhibited irregular heterogeneous surfaces with random scratch lines except for GB, where the surface roughness was more pronounced due to the use of a dental bur. The surface roughness measurement confirmed the surface topography observations in the optical images. The mean values for the average (R_a_) and peak-to-valley roughness (R_z_) of different groups are summarized in Table 2. The R_a_ and R_z_ values for the control group were 0.19±0.05 μm and 1.36±0.14 μm, respectively. Pairwise comparisons between the groups showed that R_a_ and R_z_ roughness values in GB were greater compared with the other three groups (*P* < 0.005). However, there were no significant differences in surface roughness between the control, LS, and SA groups (*P* > 0.7).

**Table 2:** Minimums, maximums, and means of surface roughness (Ra and Rz values in µm) in the four experimental groups (n = 10)

**Group**	**Minimum**	**Maximum**	**Mean**	**Standard deviation**	***P*** ** value**
Control					
Ra	0.16	0.22	0.19	0.02	< 0.005
Rz	0.74	1.03	1.36	0.14	< 0.005
GB					
Ra	1.2	2.50	1.87	0.50	< 0.005
Rz	6.29	8.08	7.46	0.71	< 0.005
LS					
Ra	0.21	0.38	0.29	0.06	< 0.005
Rz	1.61	2.52	1.90	0.37	< 0.005
SA					
Ra	0.18	0.26	0.23	0.04	< 0.005
Rz	1.19	2.13	1.6	0.37	< 0.005

*Control: no treatment; GB: grinding with diamond bur; LS: Laser treatment; SA: sandblasting with alumina.

A representative XRD pattern for a dental zirconia disk is shown in Table 3, with peaks representing tetragonal and monoclinic phases. There were significant differences in the ratio of the monoclinic phase intensity and the volumetric percentage of monoclinic phase content between different groups as a result of surface treatment (*P* < 0.001). As evident in Table 3, sandblasting and grinding treatments exhibited a significantly larger amount of monoclinic phase content (*P* < 0.001) than the laser and control groups.

**Table 3:** X-ray diffraction analysis: volumetric percentages (%) of monoclinic phase in three specimens of each experimental group

**Group**	**Minimum**	**Maximum**	**Mean**	**Standard deviation**	***P*** ** value**
Control	1.06	1.76	1.4	0.4	< 0.001
GB	4.53	4.81	4.7	0.2	< 0.001
LS	0.80	1.61	1.2	0.4	< 0.001
SA	4.27	4.33	2.9	0.0	< 0.001

*Control: no treatment; GB: grinding with diamond bur; LS: Laser treatment; SA: sandblasting with alumina

**Figure 3:**
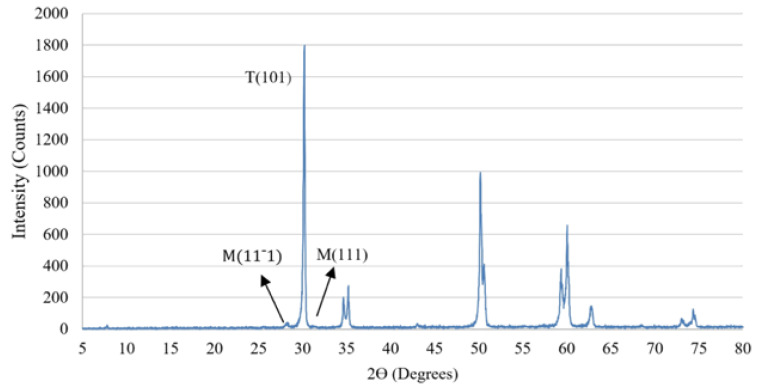
X-ray Diffraction evaluation of different surface treatment on Zirconia. Representative XRD pattern for Zirconia.

The mean biaxial flexural strengths of the control and treated samples are shown in Figure 3. Overall, there was a significant difference in the mean flexural strengths between groups (*P* < 0.01). The SA group (1023.0±74.8 MPa) exhibited a significantly higher flexural strength than only the control group (926.3±65.5 MPa) (P = 0.02) and GB (909.8±87.3 MPa) (*P* < 0.01). However, there were no significant differences between the flexural strength of the LS group (994.5±56.8 MPa) and the control, GB, and SA groups (*P* > 0.05).

**Figure 4:**
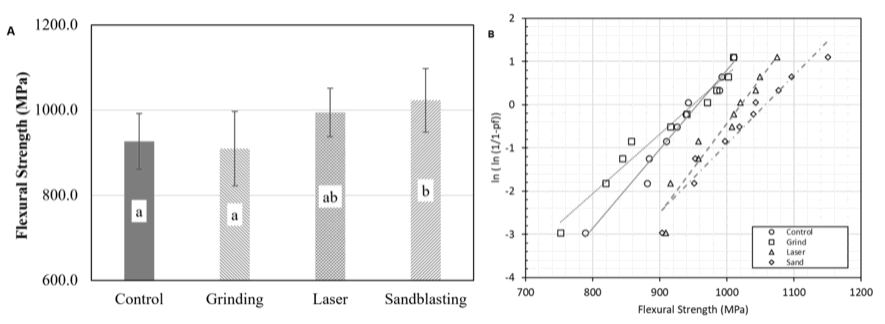
Biaxial flexural strength comparison between different surface treatment on Zirconia (A) Mechanical strength comparison (B) Weibull distribution of surface treated Zirconia strength.

Regarding the Weibull analysis, the probability of the flaw distribution and Weibull parameters are presented in Figure 4b and Table 4. The Weibull modulus (m) values for the control and SA groups were similar (around 16). The lowest Weibull modulus (m) value was for the GB group (12.2). However, the specimens in the LS group exhibited higher reliability of data with Weibull modulus calculated at 20. In terms of characteristic strength (σ_0_), the LS (1020.2 MPa) and SA groups (1023.0 MPa) exhibited similar and the highest values among the tested groups. Those values for the control and GB groups were 947.5 and 955.3 MPa, respectively.

**Table 4:** Weibull analysis of different surface treatments on zirconia

Groups*	**Weibull modulus (m)**	**Characteristic strength (σ**_0_ **)(MPa)**
Control	16.5	955.3
GB	12.2	947.5
LS	20.5	1020.2
SA	16.3	1023.0

*Control: no treatment; GB: grinding with diamond bur; LS: Laser treatment; SA: sandblasting with alumina.

Pearson’s correlation coefficient test indicated no significant relationship between the surface roughness (R_a_ and R_z_) and the biaxial flexural strength in control (*P* = 0.44), LS (P = 0.63), GB (*P* = 0.33), and SA groups (*P* = 0.98). Furthermore, there was no significant relationship between the volumetric percentage of the monoclinic phase and the flexural strength of zirconia in control (*P* = 0.560), LS (*P* = 0.516), GB (*P* = 0.632), and SA groups (*P* = 0.396).

## Discussion

This study evaluated the effects of different surface treatments on the mechanical properties of zirconia. The results showed that sandblasting, grinding with a diamond bur, and laser treatments significantly affected the surface roughness, surface topography, and flexural strength of zirconia specimens; therefore, the null hypothesis of this study was rejected.

Concerning the surface roughness and topography, the results showed that the mean R_a_ and R_z_ values for the specimens ground with a diamond bur (GB group) were significantly higher than the corresponding values in other groups (*P* < 0.005). However, there were no significant differences in the surface roughness between the control, sandblasted (SA), and laser-treated (LS) specimens (*P* > 0.05).

Overall, surface roughening is considered a crucial method to increase the bonding quality of resin cement to ceramic by introducing a micromechanical interlocking mechanism.^27^ Roughening the internal surface of ceramic restorations increases the surface area for penetration and polymerization of resin cement, leading to better adhesion.^28,29^ Both grinding and sandblasting could result in contamination removal, increased surface area, and enhanced wettability.^16,30,31^ Surface roughening by Er,Cr:YSGG laser is caused by the ablation of surface particles that can improve adhesion.^32^ In some few cases, discoloration and microcracks have been reported following laser irradiation.^17^ Extreme roughening and micro-cracks were also observed in our pilot study at higher power. However, lower power laser treatments resulted in more moderate roughness with no microcracks or discoloration on the surface (Figure 1). Recent studies showed quite different outcomes on the effectiveness of different surface treatments on zirconia,^16-18,33-36^ In a study by Martins et al,^16^ the laser treatment resulted in more surface roughness than sandblasting, and the control group exhibited the least extent of roughness. However, the present study did not show significant differences in surface roughness between the control, SA, and LS groups. The discrepancies between different studies can be attributed to different surface treatment methods and settings, such as different laser types, wavelengths, energies, sandblasting particle sizes, zirconia type, etc.

Furthermore, phase transformation in the zirconia is a crucial tool to consider to evaluate surface treatment. The stresses induced during surface roughening processes could result in phase transformation in zirconia.^37^ Surface treatment of zirconia can cause localized stress concentration and facilitate tetragonal-to-monoclinic phase transformation, which adversely affects the mechanical properties of zirconia, such as its flexural strength, hardness, and modulus of elasticity.^38^ Therefore, developing a surface treatment modality that can roughen the surface with minimal phase transformation is much desired. In this study, the mechanical surface roughening methods, including grinding and sandblasting, resulted in a greater extent of the monoclinic phase. However, surface roughness induced by Er,Cr:YSGG laser did not significantly increase the extent of the monoclinic phase on the surface. Both roughness and phase transformation can negatively influence the mechanical strength of zirconia.^39^ In this regard, sandblasted specimens exhibited higher biaxial flexural strength than grinding by a diamond bur. The laser treatment also did not show a statistically significant difference in the strength than the grinding and sandblasting methods (*P* > 0.05). However, Weibull analysis showed that the laser-treated specimens exhibited more reliable flexural strength (greater m value), implying that the distribution of flaw generated in laser treatment was more controlled and uniform.^25^

This study showed that laser could roughen the surface, while the percentage of monoclinic phase in the laser-treated specimens was significantly lower than that of sandblasted ones (*P* < 0.001) with comparable flexural strengths. These results are consistent with those of previous studies,^40-42^ which reported that zirconia’s structural integrity after laser treatment had promising durability. In this study, there were no significant differences in the flexural strength between the control, GB, and LS groups (*P* = 0.05). However, the Weibull characteristic strength and Weibull modulus showed that the GB group had lower reliability among all the other treatment methods. Furthermore, the monoclinic phase on the surfaces of the GB group increased significantly (*P* < 0.001). This finding contrasts with Kurtulmus‐Yilmaz et al,^43^ who reported superior reliability from the highest to lowest in the post-sintered grinding, post-sintered laser irradiation, and post-sintered sandblasting, respectively. On the other hand, Kosmač et al^19^ reported that the highest Weibull modulus was obtained in the control group, followed by the sandblasted and grinding treatment groups.

In the present study, the unexpected finding was for the SA and control groups. The surface roughness of the control group was comparable with the sandblasting and laser irradiation groups. This could be due to the finishing of specimens by the silicon carbide papers. In addition, while the extent of phase transformation in the sandblasted zirconia specimens (the SA group) was significantly higher than the control and LS groups, the mean flexural strength value and Weibull modulus for the SA group were significantly higher than the control group and also comparable with that of LS group. This finding contrasts with a study by Hallmann et al,^44^ where the flexural strength for the control group was higher than treatment with plasma gas, and the latter was higher than that of sandblasting with zirconia particles. It is noteworthy that the lowest flexural strength was observed after sandblasting with 150-µm alumina particles. The authors concluded that the lower flexural strength of the sandblasting group was attributed to the dominant phase of the zirconia specimens, which was identified to be the cubic phase.^44^ Similar to the present study, the laser-treated samples showed the most promising results where the flexural strength was maintained with the least phase transformation.

On the other hand, Çağlar and Yanıkoğlu^45^ reported that the flexural strengths of the sandblasted and laser-treated zirconia samples were higher than the control group. However, no significant difference in the flexural strength was observed between the sandblasted and laser groups, consistent with the present study. In general, discrepancies in the flexural results can also be attributed to numerous differences in the treatment protocols, testing configuration, zirconia types, and sample size.

Pearson’s correlation coefficient demonstrated no significant relationship between either the surface roughness or the volumetric percentage of the monoclinic phase and the biaxial flexural strength (*P* > 0.05). This finding is consistent with another study.^46^ The authors stated that despite phase transformation in the samples as a result of treatment, the mechanical performance of Yttrium-Stabilized Tetragonal Zirconia (YTZP) did not deteriorate. Perhaps, volumetric phase transformation on the surface caused by different treatments is not large enough to adversely affect zirconia’s biaxial flexural strength. Other studies have also reported that grinding, laser, and sandblasting treatments on the post-sintered samples positively affected zirconia’s flexural strength.^43,47^

Despite some promising results in some of the surface treatment methods for zirconia, the results of this study clearly showed the lack of common knowledge in the form of a standardized surface treatment method for zirconia. Finally, the effect of other variables, such as different surface treatments, different sandblasting particles, different grinding burs, different laser types and energies, aging and environmental durability, and fatigue responses, on the mechanical properties of zirconia should be evaluated further.

## Conclusion

Under the limitations of this study, the grinding of zirconia surfaces with a diamond bur resulted in high surface roughness, phase transformation, and deterioration of the flexural strength of zirconia. Sandblasting of zirconia surfaces by alumina with a great extent of phase transformation exhibited the highest flexural strength. However, a more reliable mechanical property concerning the flexural strength was obtained by the Er,Cr:YSGG laser treatment with less surface roughness and phase transformation in the zirconia. In addition, there was no significant relationship between the surface roughness or the extent of phase transformation and the biaxial flexural strength of zirconia.

## Authors’ Contributions

NY, MY, and TH were responsible for investigation and writing the original draft. SSS contributed to the concept, supervision, writing, reviewing, and editing. MAZ was responsible for formal analysis, data collection, writing, reviewing and, editing. SMRH contributed to the methodology, supervision, writing, reviewing, and editing.

## Acknowledgments

The authors would like to acknowledge Dr.* William M*. *Johnston* for his expert advice throughout this project.

## Funding

None.

## Competing Interests

None.

## Ethics Approval

Not applicable.
